# Comprehensive analysis of the prognostic and immunotherapeutic implications of STAT family members in human colorectal cancer

**DOI:** 10.3389/fgene.2022.951252

**Published:** 2022-08-19

**Authors:** Dingchang Li, Yanan Jiao, Wenxing Gao, Shidong Hu, Dingling Li, Wen Zhao, Peng Chen, Lujia Jin, Yingjie Zhao, Zhaofu Ma, Xiansheng Wu, Yang Yan, Wen Sun, Xiaohui Du, Guanglong Dong

**Affiliations:** ^1^ Medical School of Chinese PLA, Beijing, China; ^2^ Department of General Surgery, The First Medical Centre, Chinese PLA General Hospital, Beijing, China; ^3^ Medical College of Qinghai University, Xining, China; ^4^ Department of Anesthesiology, The Second Affiliated Hospital of Tianjin University of Traditional Chinese Medicine, Tianjin, China

**Keywords:** colorectal cancer, STAT transcription factors, prognostic value, immune infiltration, bioinformatics analysis, tumor immunotherapy

## Abstract

**Background:** Colorectal cancer (CRC) is the third most prevalent cancer worldwide and the second leading cause of cancer mortality. Signal transducer and activator of transcription (STAT) proteins are a group of transcription factors implicated in cell signal transduction and gene transcription in several cancer types. However, the level of expression, genetic alterations, and biological function of different STATs, as well as their prognostic and immunotherapeutic value in CRC remain unclear.

**Methods:** The mRNA and protein expression levels, genetic alterations, prognostic value, gene–gene and protein–protein interaction networks, and biological function of STATs in CRC were studied using the GEPIA, HPA, cBioPortal, PrognoScan, Kaplan–Meier plotter, GeneMANIA, STRING, and Metascape databases. The expression of STATs in CRC was confirmed using immunohistochemistry (IHC). Finally, the relationship between STAT expression and immune infiltration as well as immunotherapy-associated indicators was also investigated.

**Results:** The expression levels of *STAT2/5A/5B* are downregulated in CRC, and the *STAT1/3/4/5B* expressions were significantly associated with the tumor stage of patients with CRC. The abnormal expression of *STAT2/4/5B* in patients with CRC is related to the prognosis of patients with CRC. The STATs and their neighboring proteins are primarily associated with lymphocyte activation, cytokine-mediated signaling pathways, positive regulation of immune response, regulation of cytokine production, and growth hormone receptor signaling pathways in cancer. The expression of STATs was significantly associated with immune infiltration and immunotherapy response-associated indicators.

**Conclusion:** This study may help further understand the molecular mechanism of CRC and provide new prognostic biomarkers and immunotherapy targets in patients with CRC.

## Introduction

Global cancer data in 2020 showed that colorectal cancer (CRC) was the third most prevalent cancer globally and the second leading cause of cancer mortality (Sung et al., 2021). Since the early symptoms of colorectal cancer are not typical (Yoshioka et al., 2014), 35% of patients are often found with metastatic disease when they are diagnosed, and 50% of patients without metastasis ultimately develop metastatic CRC (Zacharakis et al., 2010). Despite advances in chemotherapy, targeted therapy, and immunotherapy, the clinical outcome of CRC remains poor, especially in metastatic CRC (Brenner et al., 2014). Thus, exploring the possible pathogenetic mechanisms of CRC, as well as discovering early diagnostic biomarkers and treatment targets, is crucial for improving patients’ prognoses.

STATs are a group of transcription factors encoded by seven members (*STAT1/2/3/4/5A/5B/6*) of the STAT gene family that are involved in cell proliferation, differentiation, apoptosis, and immune system regulation (Verhoeven et al., 2020). As a result, dysregulation of their pathway would result in a variety of diseases, including cancer (Bowman et al., 2000). Extensive studies have already demonstrated that inappropriate activation of specific STAT members contributes to oncogenesis, especially for the Janus kinase (JAK)/*STAT3* pathway, which has been linked to many types of cancer (Johnson et al., 2018). For example, Li et al. reported that long non-coding RNA RP11-468E2.5 could curtail CRC development and promote apoptosis via the JAK/STAT signaling pathway by targeting *STAT5* and *STAT6* (Jiang et al., 2019).

Despite great importance of STATs in malignancies, there has been no study to explore the implications of every STAT factor in CRC, including their expression level, genetic variation, biological function, and potential molecular mechanism. Furthermore, their correlation with the prognosis, immune infiltration, and immunotherapy response in patients with CRC also remains unknown. Thus, it is necessary to comprehensively analyze the significance of each STAT member in CRC development and progression.

Multiple large-scale bioinformatics databases were used in this study for comprehensive bioinformatics analysis of the expression of STATs and their associations with tumor stage in patients with CRC. In addition, immunohistochemistry (IHC) was used to confirm the differential expressions of STATs in CRC and normal tissues. Subsequently, the genetic variation, biological function, and molecular mechanism of each STAT member in CRC were explored. Ultimately, the relationship between the expression of STATs and prognosis, immune infiltration, and immunotherapy response in patients with CRC was analyzed.

## Materials and methods

### Data acquisition and analysis of differential expression

The Genotype-Tissue Expression (GTEx) database (https://commonfund.nih.gov/GTEx/) collects data from 54 normal human tissues for sequencing, which can be used to compare the differential level of gene expression between normal and diseased tissues (GTEx Consortium, 2013). The Cancer Genome Atlas (TCGA) (https://tcga.xenahubs.net) mainly contains data from 33 different types of tumors. The RNA sequencing data of normal samples from the GTEx database and tumor samples from TCGA were downloaded, and the Wilcoxon rank-sum test method was used to compare the differential mRNA expressions of STATs between 33 different types of cancers and corresponding normal tissues. Threshold values were determined according to the following values: ns, *p* ≥ 0.05, *p < 0.05, **p < 0.01, and ***p < 0.001. The “ggplot2” R package was used for the boxplot.

### Gene Expression Profiling Interactive Analysis 2 (GEPIA2) dataset

GEPIA2 (http://gepia2.cancer-pku.cn) is the latest version of GEPIA, which analyzes RNA sequencing expression data including 9,736 tumors and 8,587 normal samples from TCGA and GTEx projects using standard processing pipelines (Tang et al., 2019). GEPIA2 offers a variety of functions such as differential gene expression analysis, cancer types and pathological staging, similar gene detection, patient survival analysis, correlation analysis, and dimensionality reduction analysis.

### Human Protein Atlas (HPA) dataset and immunohistochemistry (IHC)

HPA database (https://www.proteinatlas.org/) was used to compare the STAT gene protein expression in the CRC tissues and the corresponding normal tissues.

IHC staining was used to further validate the reliability of the above results. Clinical samples were collected from 21 patients with CRC who were undergoing surgical treatment in our hospital, and their clinical information is shown in Supplementary Table S1. Firstly, these samples were made into 3 μm paraffin sections and incubated with rabbit monoclonal antibodies of *STAT1*, *STAT2*, *STAT3*, *STAT4*, *STAT5A*, *STAT5B*, and *STAT6* (1:100, all from Abcam, USA) at 4°C overnight. The sections were then conjugated with horseradish peroxidase (HRP) secondary antibody (Abcam, USA) at 1/500 dilution at room temperature for 2 h. Subsequently, the conjugates were stained with 3,3′-diaminobenzidine (DAB) reagent, and ultimately counterstained with hematoxylin. The IHC score of STATs was assessed manually and calculated by a pathologist. The percentage of positive cells was scored as: 0 (0–10% positive); 1 (10–25% positive); 2 (26–50% positive); 3 (51–75% positive); and 4 (≥76% positive). The staining intensity was scored as: 0 (no staining); 1 (weak); 2 (moderate); and 3 (strong). The overall IHC score was calculated by multiplying the score of positive cells (0–4) by the staining intensity (0–3).

### Tumor–Immune System Interactions and Drug Bank (TISIDB) database

TISIDB (http://cis.hku.hk/TISIDB) is a website for the tumor and immune system interaction that integrates multiple types of data in oncoimmunology and reports genes related to antitumor immunity, tumor cell resistance or sensitivity to T cell-mediated killing and immunotherapy, and relationships between genes and immune features of 30 cancer types from TCGA (Ru et al., 2019).

### PrognoScan database and the Kaplan–Meier plotter analysis

The prognostic value of STATs mRNA expression in patients with CRC was assessed by the PrognoScan Database (http://www.abren.net/PrognoScan/) (Mizuno et al., 2009). This could be used for evaluating the correlation between gene expression and patient survival including overall survival (OS) and disease-free survival (DFS). Cox *p* < 0.05 was considered statistically significant.

The Kaplan–Meier plotter (www.kmplot.com), an online database containing gene expression data and clinical survival information of cancer patients (Nagy et al., 2018), was further used to validate the relationship between STAT expression in rectal adenocarcinoma (READ) and OS. The hazard ratio (HR) with 95% confidence intervals and log-rank *p*-value were also calculated.

### cBioPortal

cBioPortal (http://www.cbioportal.org) is an online database that can conduct multidimensional cancer genomics studies (Gao et al., 2013). A colorectal adenocarcinoma dataset (TCGA, PanCancer Atlas) containing 524 patients was selected to analyze the expression of STATs. The genomic profiles included mutations, putative copy-number alterations from Genomic Identification of Significant Targets in Cancer (GISTIC) scores, and mRNA expression z-scores (RNA Seq V2 RSEM). The z-score threshold was set at ±1.8.

### Network analysis

GeneMANIA (www.genemania.org), an online analysis tool that provides protein and genetic co-expression, co-localization, interactions, pathways, and shared protein domains of submitted genes (Franz et al., 2018), was used to perform a gene–gene interaction network for STATs. STRING (https://string-db.org), an online dataset that collects and integrates all publicly available protein–protein interaction (PPI) data and predicts potential functions (Szklarczyk et al., 2019), was used to construct a PPI network for STATs.

### Functional enrichment analysis

Firstly, GEPIA was used to identify the top 30 similar genes in CRC for each STAT family member. Metascape was subsequently used to perform Gene Ontology (GO) and Kyoto Encyclopedia of Genes and Genomes (KEGG) enrichment pathway analysis of the STATs and similar genes (Zhou et al., 2019). Only terms with *p* < 0.01, minimum count >3, and enrichment factor >1.5 were considered significant.

### Tumor Immune Estimation Resource (TIMER) dataset

TIMER (http://timer.cistrome.org/), an online dataset that provides tumor immune infiltrating abundances estimated by multiple immune deconvolution methods (Li et al., 2020), was used in this study to evaluate the correlation between STAT expression levels and immune cell infiltration.

### Statistical analysis

The difference between STAT IHC scores in normal and tumor tissues was tested using a two-tailed Student’s t-test with unpaired analysis. The correlation between STAT gene expressions and immune infiltration level, tumor purity, immune checkpoints, tumor mutation burden (TMB), microsatellite instability (MSI), and mismatch repair (MMR) genes in CRC was assessed using Spearman’s correlation coefficients, and a *p* < 0.05 was considered statistically significant.

## Results

### The expression levels of STATs in pan-cancer

**FIGURE 1:**
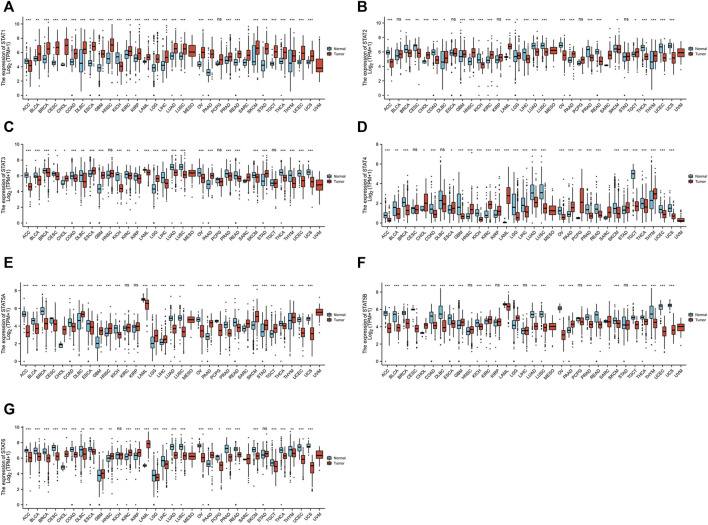
Transcription levels of signal transducer and activator of transcription (STAT) factors in different types of cancers from The Cancer Genome Atlas (TCGA) database and the Genotype-Tissue Expression (GTEx) database.

STAT expression levels in 33 types of tumors were evaluated using data from TCGA database (n = 9,379) and GTEx database (n = 8,293). In most types of tumors, all STAT family members had significantly abnormal levels of expression compared to normal tissues (Figure 1). Tumors of the digestive system were concentrated on since this study is mainly about CRC. The *STAT1* gene was highly expressed in most tumors, including cholangiocarcinoma (CHOL), colon adenocarcinoma (COAD), esophageal carcinoma (ESCA), liver hepatocellular carcinoma (LIHC), pancreatic adenocarcinoma (PAAD), READ, and stomach adenocarcinoma (STAD) (Figure 1A). The *STAT2* gene expression levels were high in CHOL and PAAD but low in COAD and READ (Figure 1B). The *STAT3* gene was highly expressed in CHOL, ESCA, PAAD, and STAD but not in COAD, LIHC, or READ (Figure 1C). The expression of *STAT4* was high in CHOL, ESCA, PAAD, and STAD but low in COAD, LIHC, and READ (Figure 1D). The *STAT5A* gene expression was high in CHOL, LIHC, PAAD, and STAD but low in COAD, ESCA, and READ (Figure 1E). The expression of *STAT5B* was high in CHOL and PAAD but low in COAD, ESCA, and READ (Figure 1F). *STAT6* presented high expression in CHOL and PAAD but low expression in COAD, ESCA, LIHC, and READ (Figure 1G).

### Transcriptional and translational expression levels of STATs in CRC patients

**FIGURE 2:**
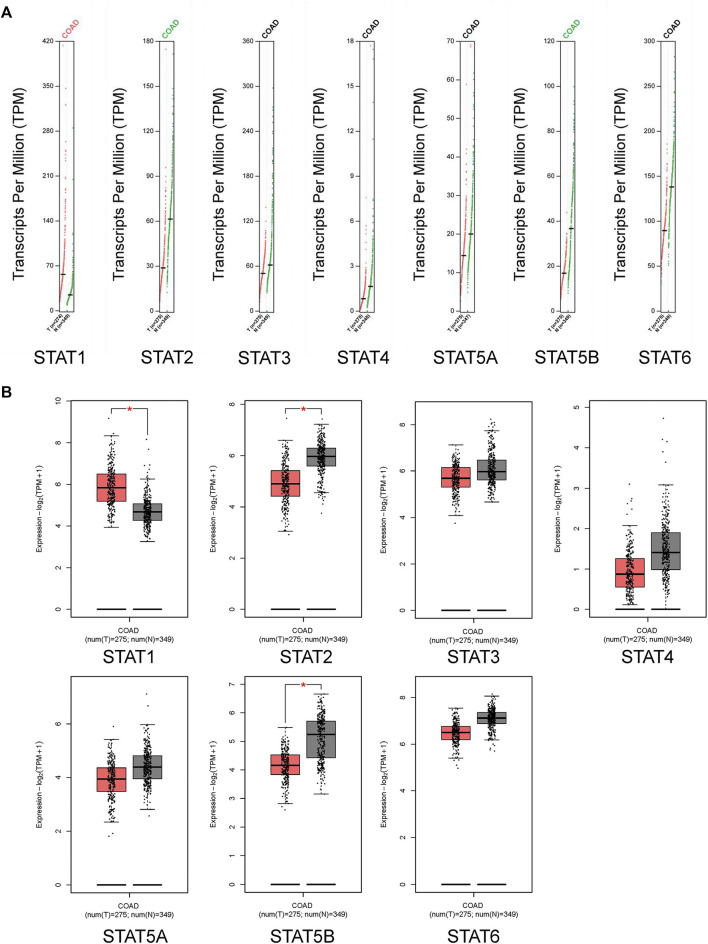
Transcription levels of signal transducer and activator of transcription (STAT) factors in colorectal cancer (CRC) from the Gene Expression Profiling Interactive Analysis 2 (GEPIA2) dataset.

The GEPIA dataset was used to compare the transcriptional levels of STATs between CRC and normal tissues (Figure 2A and Figure 2B). The results showed that the expression level of *STAT1* in CRC tissues was higher than in normal colon tissues, and the transcriptional levels of *STAT2* and *STAT5B* in CRC were lower than in normal tissues significantly. The expressions of *STAT3/4/5A/6* genes were lower in CRC than in normal samples, although there was no statistical significance.

**FIGURE 3:**
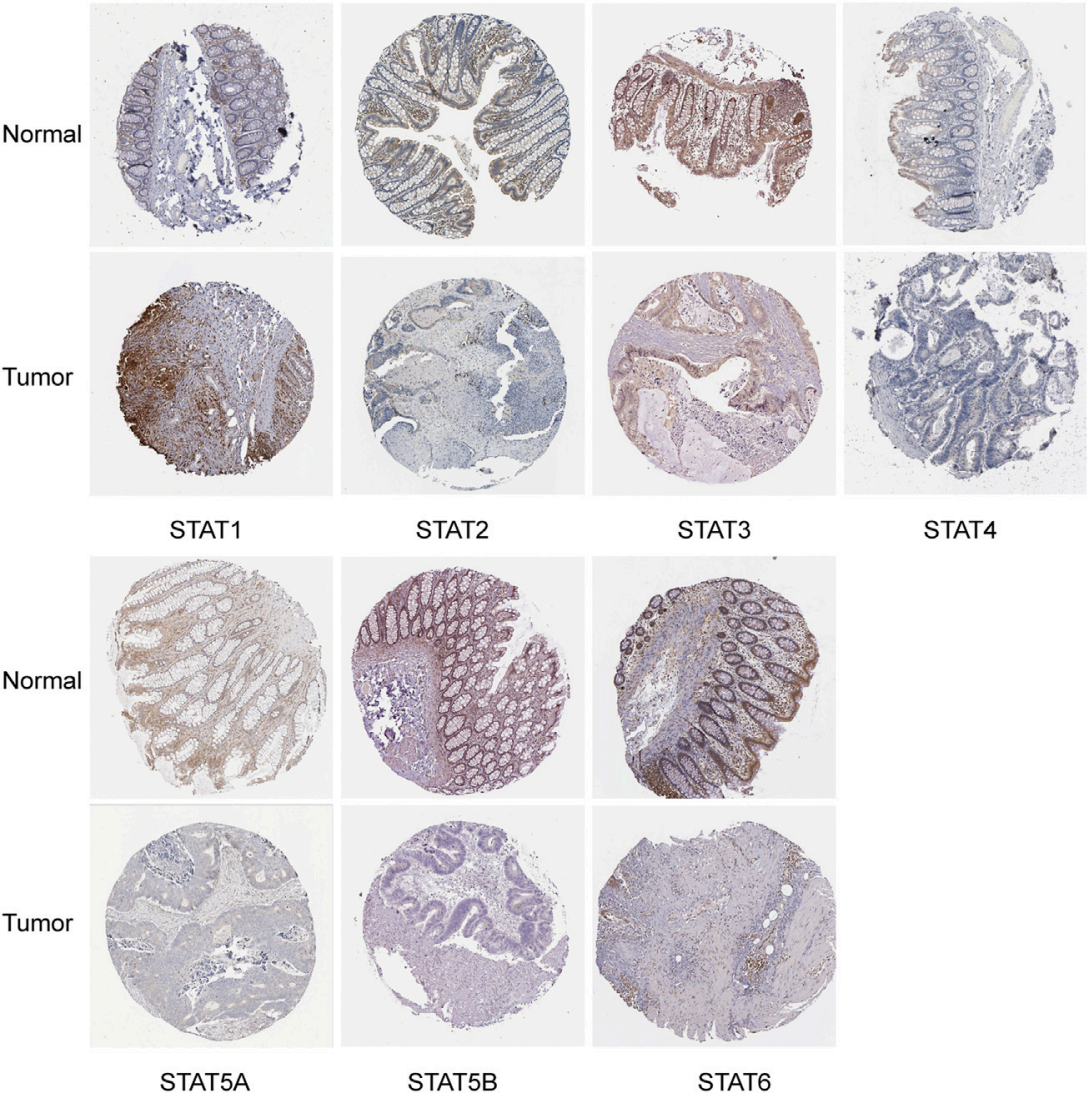
Translation levels of signal transducer and activator of transcription (STAT) factors in colorectal cancer (CRC) from the Human Protein Atlas (HPA) dataset.

**FIGURE 4:**
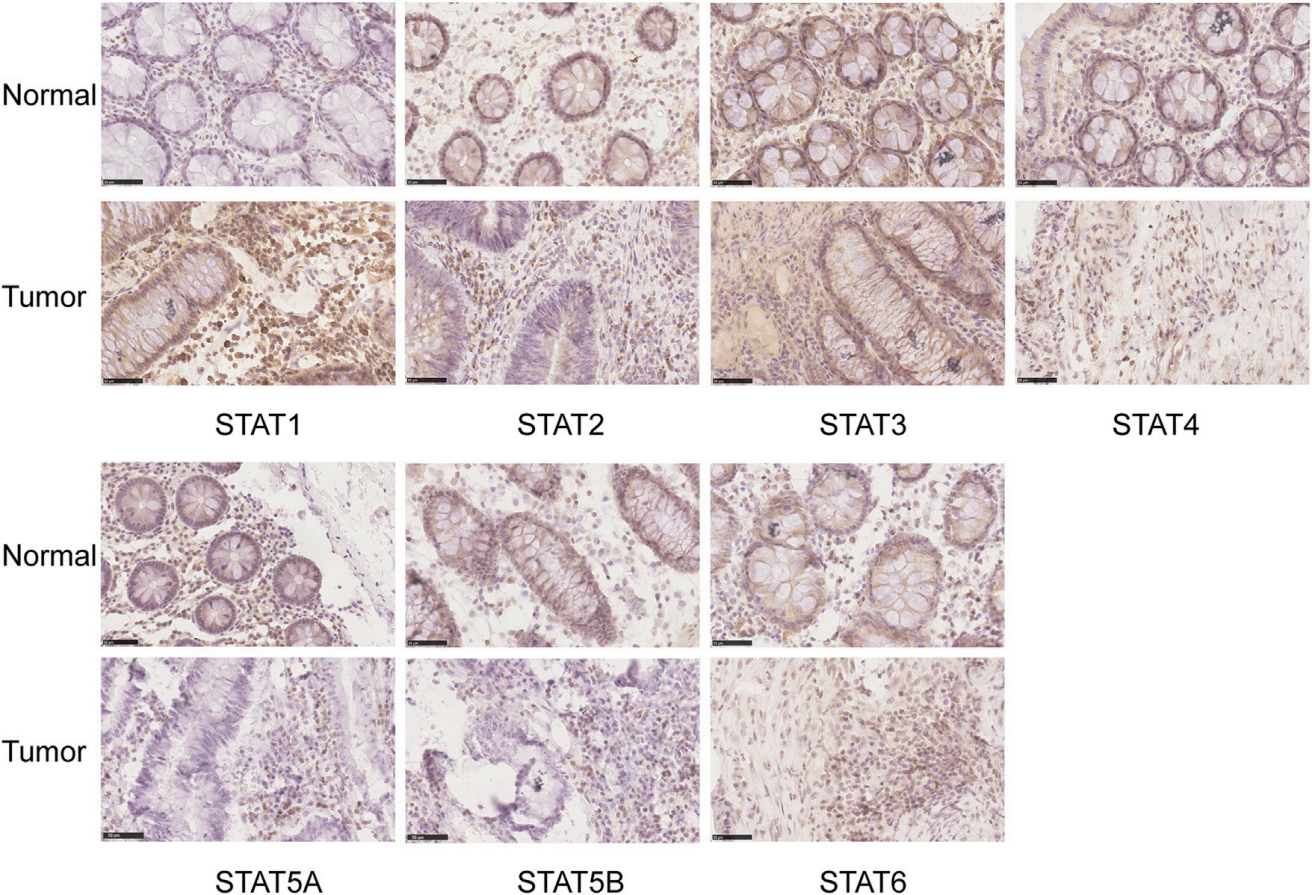
Translation levels of signal transducer and activator of transcription (STAT) factors in colorectal cancer (CRC) with immunohistochemistry (IHC). Scale bar = 50 µm.

The HPA database and IHC staining were used to further confirm the protein expression of STATs in CRC and normal tissues. The HPA database results indicated that the *STAT1* protein was more highly expressed in the CRC tissues than in the normal tissues, while *STAT2/5A/5B/6* were significantly less expressed in CRC tissues than in normal tissues (Figure 3). The IHC results and scores from clinical samples showed the protein levels of *STAT1* were higher, and levels of *STAT2/5A/5B* were lower in CRC tissues than in the adjacent normal tissues with great significance (Figure 4 and Supplementary Figure S1).

### The prognostic value of STATs in CRC patients

**FIGURE 5:**
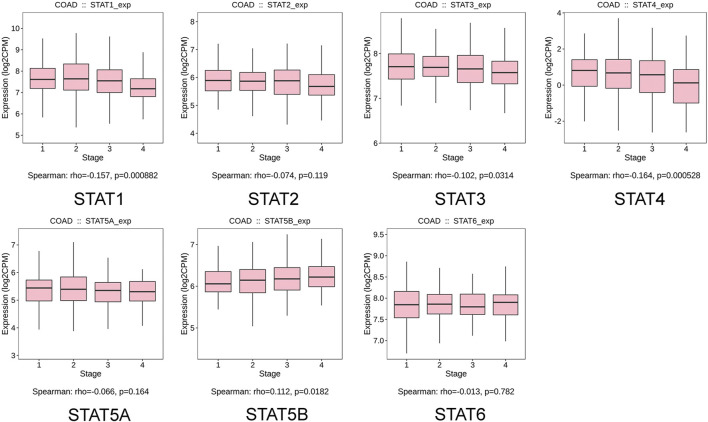
Correlation between signal transducer and activator of transcription (STAT) factor expression and tumor stage in patients with colorectal cancer (CRC) from the Tumor–Immune System Interactions and Drug Bank (TISIDB) database.

The relationship between transcriptional levels of STATs and CRC stage was investigated using the TISIDB. The results showed that the expression levels of *STAT1/3/4/5B* were significantly associated with the tumor stage of patients with CRC. However, there was no significant correlation between the *STAT2/5A/6* expression and tumor stage (Figure 5).

**FIGURE 6:**
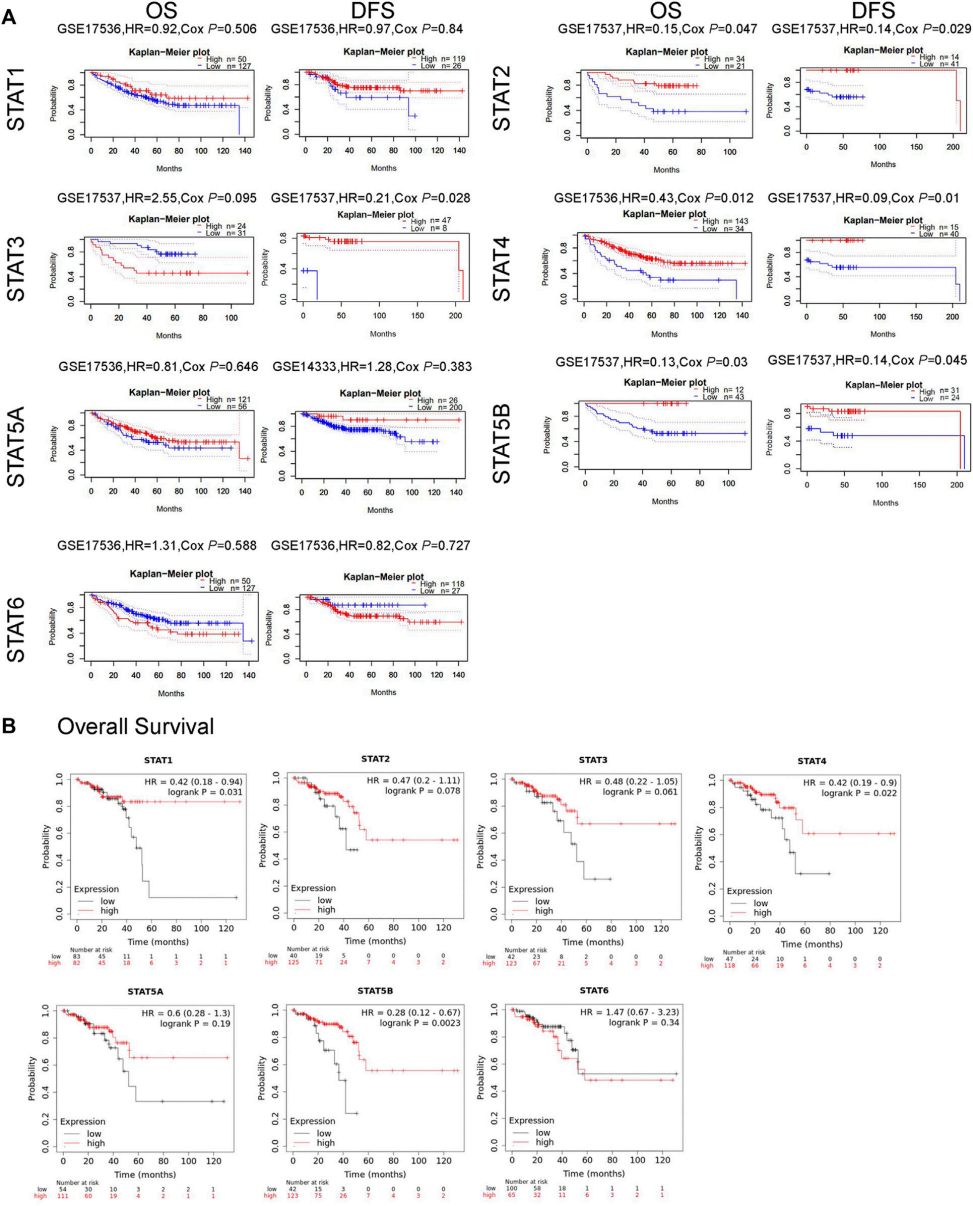
Relationship between signal transducer and activator of transcription (STAT) factor expression and prognosis in patients with colorectal cancer (CRC). **(A)** Prognostic value of STATs in patients with CRC in the OS and DFS curves (PrognoScan). **(B)** Prognostic value of STATs in rectal adenocarcinoma (READ) patients in the OS curve (Kaplan–Meier plotter).

The correlation between STAT expression and clinical outcome was evaluated using the PrognoScan database and the Kaplan–Meier plotter analysis to assess the value of STATs expression levels in the prognosis of CRC (Figure 6). The PrognoScan database analysis results showed that higher *STAT2/4/5B* mRNA levels were significantly associated with better OS (*p* < 0.05) and increased *STAT2/3/4/5B* transcription levels were significantly associated with longer DFS (*p* < 0.05) (Figure 6A). In addition, the correlation between STAT expression levels and OS in patients with READ was further validated using the Kaplan–Meier plotter, which indicated that high expression of *STAT1/4/5B* favored OS (*p* < 0.05) (Figure 6B).

### Gene mutations, co-expression, and interaction analyses of STATs in CRC patients

**FIGURE 7:**
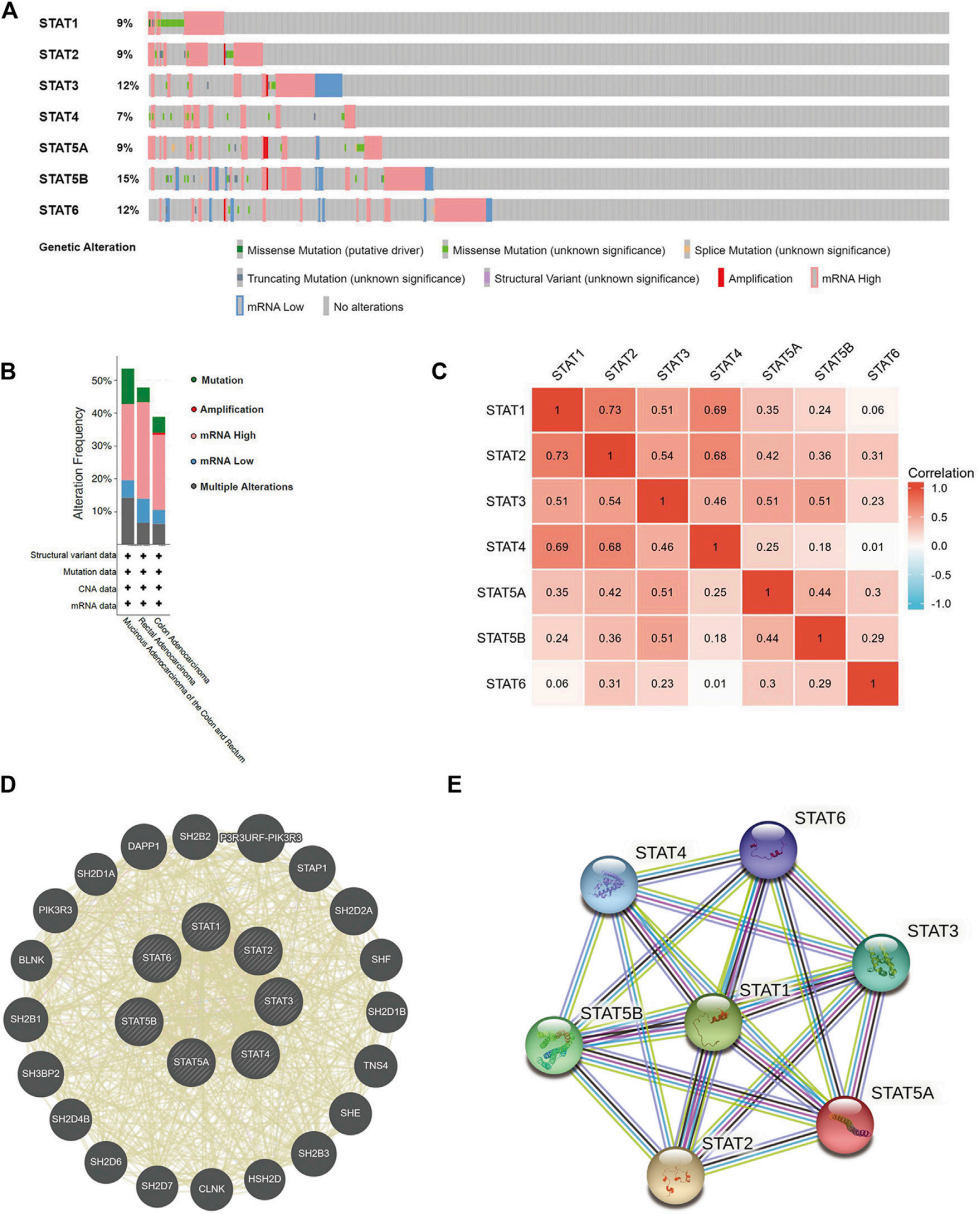
Genetic mutations, co-expression, and interaction analysis of signal transducer and activator of transcription (STAT) factors at gene and protein levels in patients with colorectal cancer (CRC) (cBioPortal, GeneMANIA, and STRING). **(A)** Analysis of gene mutations of STAT family members in CRC. **(B)** Summary of alterations in expressed STATs in CRC. **(C)** Correlation heatmap of expressed STATs in CRC. The numbers in the color blocks represent Spearman’s correlation coefficient. **(D)** Gene–gene interaction network among STATs predicted by GeneMANIA. **(E)** Protein–protein interaction network among STATs predicted by STRING.

The cBioPortal online tool was used to evaluate genetic alterations and STAT factor correlations in patients with CRC. STATs were found to be altered in 224 (43%) of 524 patients (Figure 7A). The genes with the highest and lowest mutation rates in STATs are *STAT5B* (15%) and *STAT4* (7%), respectively. The others are *STAT1* (9%), *STAT2* (9%), *STAT3* (12%), *STAT5A* (9%), and *STAT6* (12%) (Figure 7A). It was also found that patients with colorectal mucinous adenocarcinoma were most likely to have STAT gene alterations (53.57% of 56 cases) (Figure 7B). The analysis results from cBioPortal showed that patients in the unaltered group seemed /to have a better prognosis than those in the altered group but without statistical significance (Supplementary Figure S2A). The potential effects of every single STAT factor on prognosis were then evaluated and the results showed that patients with altered *STAT4* had significantly poorer prognostic outcomes compared with unaltered patients (Supplementary Figure S2E). TIMER dataset analysis was used to evaluate the effect of STAT mutations on five types of immune cell infiltration, and the outcomes indicated that mutated *STAT1* correlates with a higher level of neutrophil infiltration; mutated *STAT4* correlates with more B cells, CD8^+^ T cells, and neutrophil infiltration; mutated *STAT5A* correlates with a higher level of CD4^+^ T cells and neutrophils; mutated *STAT5B* correlates with more B cells and; mutated *STAT6* correlates with lower B cell infiltration with significance (Supplementary Figure S3).

We further explored the co-expression of STAT members in patients with CRC, and there were strong or moderate positive relationships between *STAT1* and *STAT2*, *STAT3*, *STAT4*, and *STAT5A*; *STAT2* and *STAT3*, *STAT4*, *STAT5A*, *STAT5B*, and *STAT6*; *STAT3* and *STAT4*, *STAT5A*, and *STAT5B* and; *STAT5A* and *STAT5B* and *STAT6* (*p* < 0.05) (Figure 7C).

The gene–gene interaction (GGI) network of STATs was established using the GeneMANIA database (Figure 7D). Based on shared protein domains, co-localization, physical interactions, co-expression, pathways, and genetic interactions, 20 related genes were enriched in this network. These genes are involved in a variety of functions such as receptor tyrosine kinase binding, receptor signaling pathway via STAT, signaling receptor complex adaptor activity, signaling adaptor activity, phosphoprotein binding, protein phosphorylated amino acid binding, and growth hormone receptor signaling pathway. STRING was used to explore the potential interactions between STATs at the protein level, as shown in Figure 7E, where the PPI network diagram had seven nodes and 21 edges. The analysis results of PPI network indicated that each STAT factor has known or predicted interactions with the others, especially *STAT3*, which has experimentally determined interactions with each of the other factors. The top four molecular pairs with strong functional links based on combined scores were *STAT1* and *STAT2*, *STAT1* and *STAT3*, *STAT3* and *STAT5B*, and *STAT1* and *STAT5A*.

### Functional enrichment analysis of STATs and the genes similar to them in CRC patients

**FIGURE 8:**
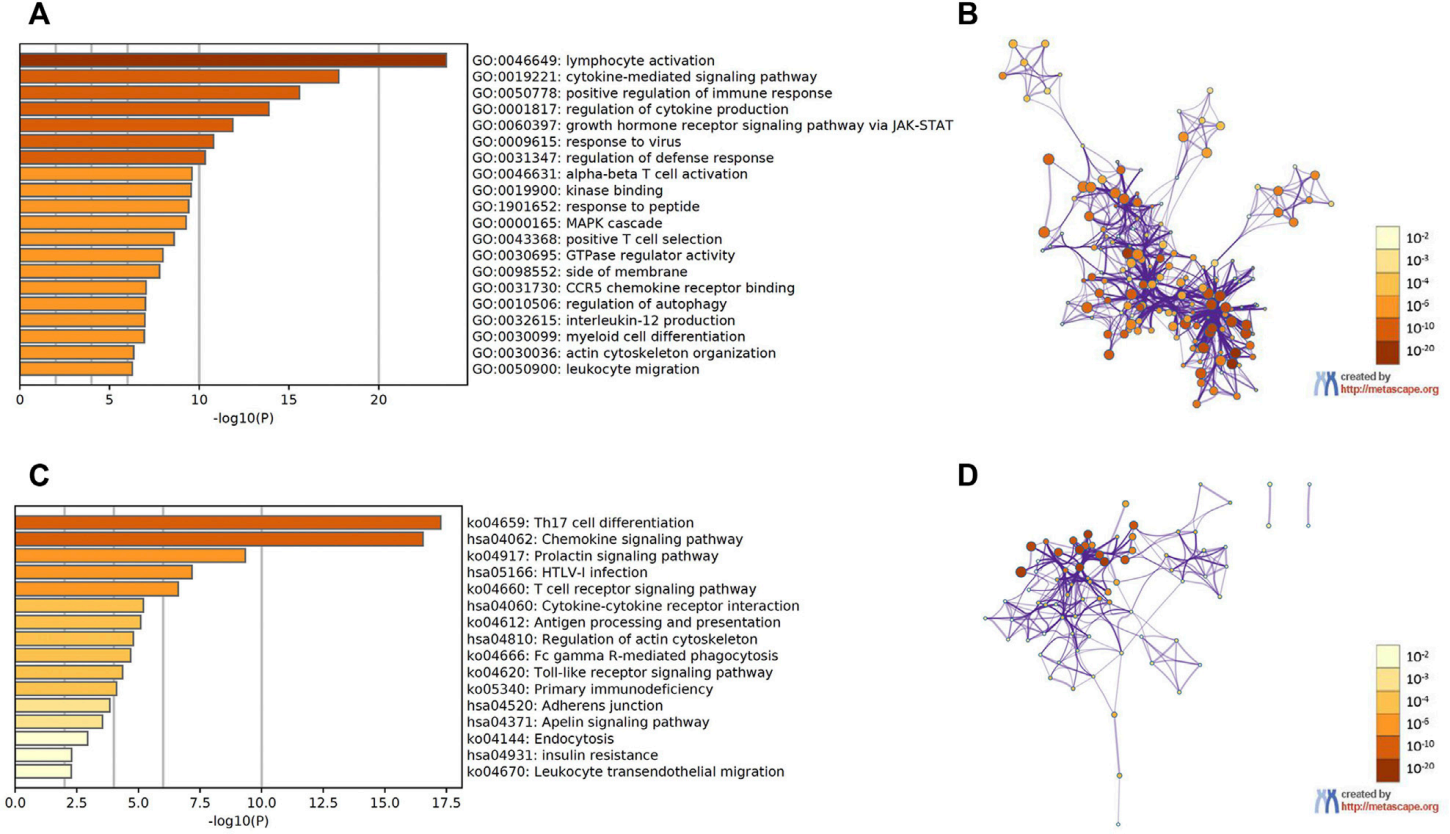
Functional enrichment analysis of signal transducer and activator of transcription (STAT) factors and the genes similar to them in patients with colorectal cancer (CRC) (Metascape). **(A)** Heatmap of GO enriched terms colored by *p*-values. **(B)** Network of GO enriched terms colored by *p*-value, where terms containing more genes tend to have a more significant *p*-value. **(C)** Heatmap of KEGG enriched terms colored by *p*-values. **(D)** Network of KEGG enriched terms colored by *p*-value, where terms containing more genes tend to have a more significant *p*-value.

GEPIA2 datasets were used to identify the top 30 genes that have a similar expression pattern to each STAT family member. The GO and KEGG enrichment pathway analyses of STATs and their similar genes were then performed using Metascape. The top 20 GO enrichment items were composed of 16 biological processes (BP) items, three molecular functions (MF) items, and one cellular component (CC) item (Figure 8A, Figure 8B, and Table 1). The first five projects are all in the BPs, and they are lymphocyte activation, cytokine-mediated signaling pathway, positive regulation of immune response, regulation of cytokine production, and growth hormone receptor signaling pathway via JAK/STAT. MFs that were significantly related to STATs and similar genes were kinase binding, GTPase regulator activity, and CCR5 binding. The only one CC was side of the membrane.

**TABLE 1:** Gene Ontology (GO) functional enrichment analysis of signal transducer and transcription (STAT) factors and neighbor genes in colorectal cancer (CRC) (Metascape).

GO	Category	Description	Count	%	Log10 (P)	Log10 (q)
GO:0046649	GO biological processes	Lymphocyte activation	39	21.08	-23.76	-19.41
GO:0019221	GO biological processes	Cytokine-mediated signaling pathway	27	14.59	-17.78	-13.90
GO:0050778	GO biological processes	Positive regulation of immune response	27	14.59	-15.59	-11.84
GO:0001817	GO biological processes	Regulation of cytokine production	29	15.68	-13.88	-10.49
GO:0060397	GO biological processes	Growth hormone receptor signaling pathway via JAK/STAT	6	3.24	-11.87	-8.86
GO:0009615	GO biological processes	Response to virus	18	9.73	-10.80	-8.00
GO:0031347	GO biological processes	Regulation of defense response	22	11.89	-10.32	-7.56
GO:0046631	GO biological processes	Alpha-beta T-cell activation	12	6.49	-9.60	-6.96
GO:0019900	GO molecular functions	Kinase binding	23	12.43	-9.55	-6.93
GO:1901652	GO biological processes	Response to peptide	19	10.27	-9.41	-6.79
GO:0000165	GO biological processes	MAPK cascade	23	12.43	-9.27	-6.67
GO:0043368	GO biological processes	Positive T-cell selection	7	3.78	-8.60	-6.06
GO:0030695	GO molecular functions	GTPase regulator activity	17	9.19	-7.98	-5.50
GO:0098552	GO cellular components	Side of membrane	19	10.27	-7.79	-5.34
GO:0031730	GO molecular functions	CCR5 binding	4	2.16	-7.03	-4.64
GO:0010506	GO biological processes	Regulation of autophagy	13	7.03	-7.00	-4.62
GO:0032615	GO biological processes	Interleukin-12 production	7	3.78	-6.98	-4.61
GO:0030099	GO biological processes	Myeloid cell differentiation	14	7.57	-6.94	-4.57
GO:0030036	GO biological processes	Actin cytoskeleton organization	18	9.73	-6.35	-4.06
GO:0050900	GO biological processes	Leukocyte migration	13	7.03	-6.27	-3.99

The first 16 KEGG pathways are displayed in Figure 8C, Figure 8D, and Table 2. The results indicated the involvement of STATs in pathways such as Th17 cell differentiation, chemokine signaling pathway, T cell receptor signaling pathway, and cytokine–cytokine receptor interaction.

**TABLE 2:** Kyoto Encyclopedia of Genes and Genomes (KEGG) functional enrichment analysis of signal transducer and transcription (STAT) factors and neighbor genes in colorectal cancer (CRC) (Metascape).

GO	Category	Description	Count	%	Log10 (P)	Log10 (q)
ko04659	KEGG pathway	Th17 cell differentiation	16	8.65	-17.27	-14.39
hsa04062	KEGG pathway	Chemokine signaling pathway	19	10.27	-16.55	-14.25
ko04917	KEGG pathway	Prolactin signaling pathway	9	4.86	-9.33	-7.76
hsa05166	KEGG pathway	HTLV-I infection	12	6.49	-7.17	-5.78
ko04660	KEGG pathway	T-cell receptor signaling pathway	8	4.32	-6.61	-5.26
hsa04060	KEGG pathway	Cytokine–cytokine receptor interaction	11	5.95	-5.21	-3.96
ko04612	KEGG pathway	Antigen processing and presentation	6	3.24	-5.10	-3.87
hsa04810	KEGG pathway	Regulation of actin cytoskeleton	9	4.86	-4.79	-3.62
ko04666	KEGG pathway	Fc gamma R-mediated phagocytosis	6	3.24	-4.68	-3.53
ko04620	KEGG pathway	Toll-like receptor signaling pathway	6	3.24	-4.35	-3.23
ko05340	KEGG pathway	Primary immunodeficiency	4	2.16	-4.12	-3.04
hsa04520	KEGG pathway	Adherens junction	5	2.70	-3.85	-2.79
hsa04371	KEGG pathway	Apelin signaling pathway	6	3.24	-3.54	-2.54
ko04144	KEGG pathway	Endocytosis	7	3.78	-2.94	-2.01
hsa04931	KEGG pathway	Insulin resistance	4	2.16	-2.28	-1.49
ko04670	KEGG pathway	Leukocyte transendothelial migration	4	2.16	-2.27	-1.48

### The relationship between STAT expression levels and immune infiltration levels in CRC

According to the results of GO and KEGG enrichment analysis, it was found that STATs were closely related to immune functions such as lymphocyte activation, positive regulation of immune response, Th17 cell differentiation, and T cell receptor signaling pathway. The results indicated that STATs were involved in the regulation of the tumor immune microenvironment, which is closely related to the initiation and progression of tumors.

**FIGURE 9:**
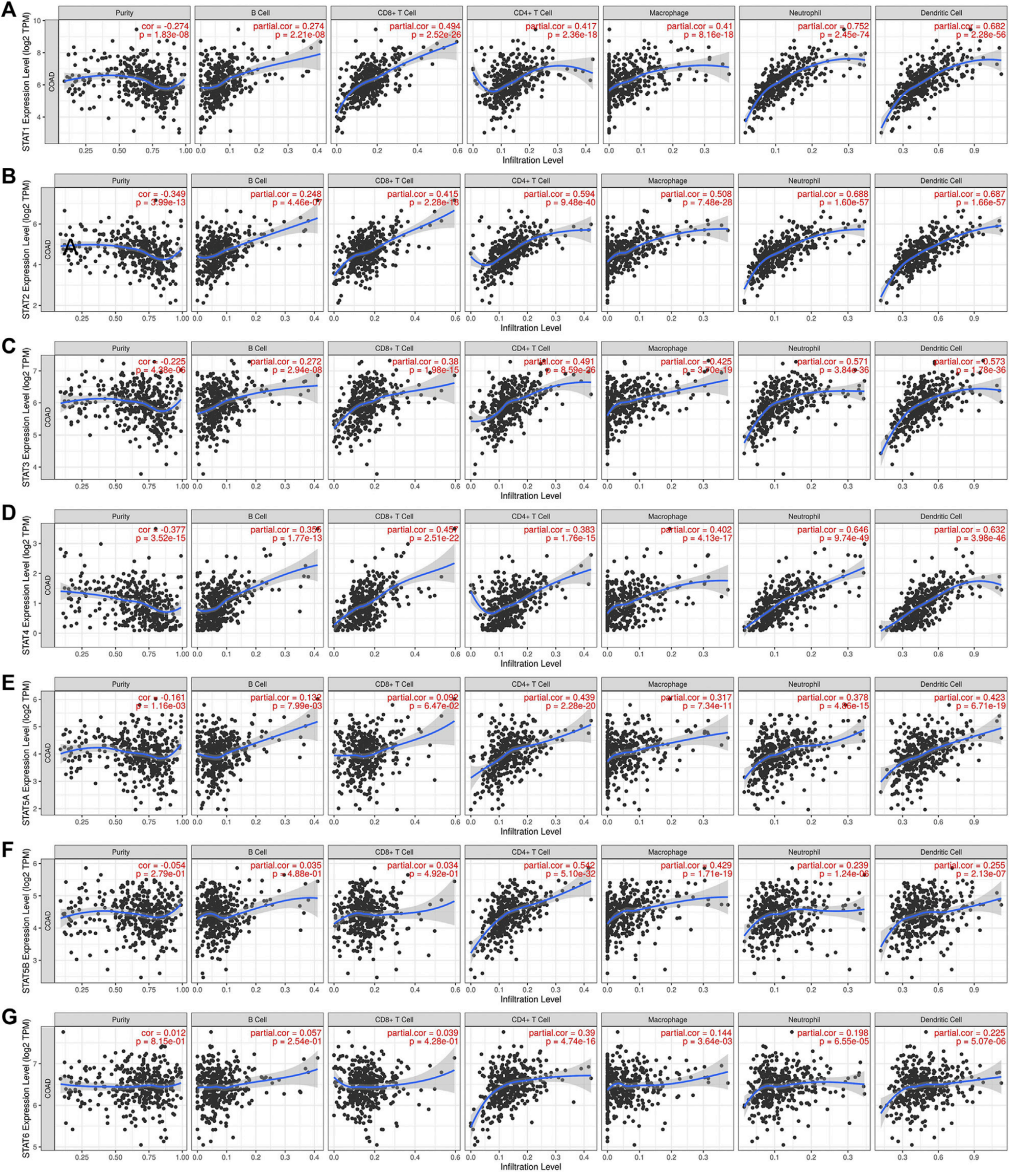
Correlation between signal transducer and activator of transcription (STAT) factor expression levels and immune infiltration levels in colorectal cancer (CRC) from the Tumor Immune Estimation Resource (TIMER) dataset. The relationship between the abundance of immune cells and the expression of **(A)** *STAT1*, **(B)** *STAT2*, **(C)** *STAT3*, **(D)** *STAT4*, **(E)** *STAT5A*, **(F)** *STAT5B*, and **(G)** *STAT6* in CRC.

As a result, we used TIMER online dataset to evaluate the relationship between STAT expression and immune cell infiltration in CRC. As shown in Figure 9, the outcomes, as expected, revealed that STATs were involved in many types of immune cell infiltration and influenced the clinical outcome of patients with CRC. *STAT1/2/3/4* expressions had a positive correlation with the infiltration of B cells, CD8^+^ T cells, CD4^+^ T cells, macrophage, neutrophil, and dendritic cells (Figures 9A–D). The expressions of *STAT5A/5B/6* were positively correlated with the infiltration of CD4^+^ T cells, macrophage, neutrophil, and dendritic cells, and *STAT5A* expression was also positively related to the infiltration of B cells (Figures 9E–G).

### The correlation between STATs and immunotherapy response-related indicators in CRC

**FIGURE 10:**
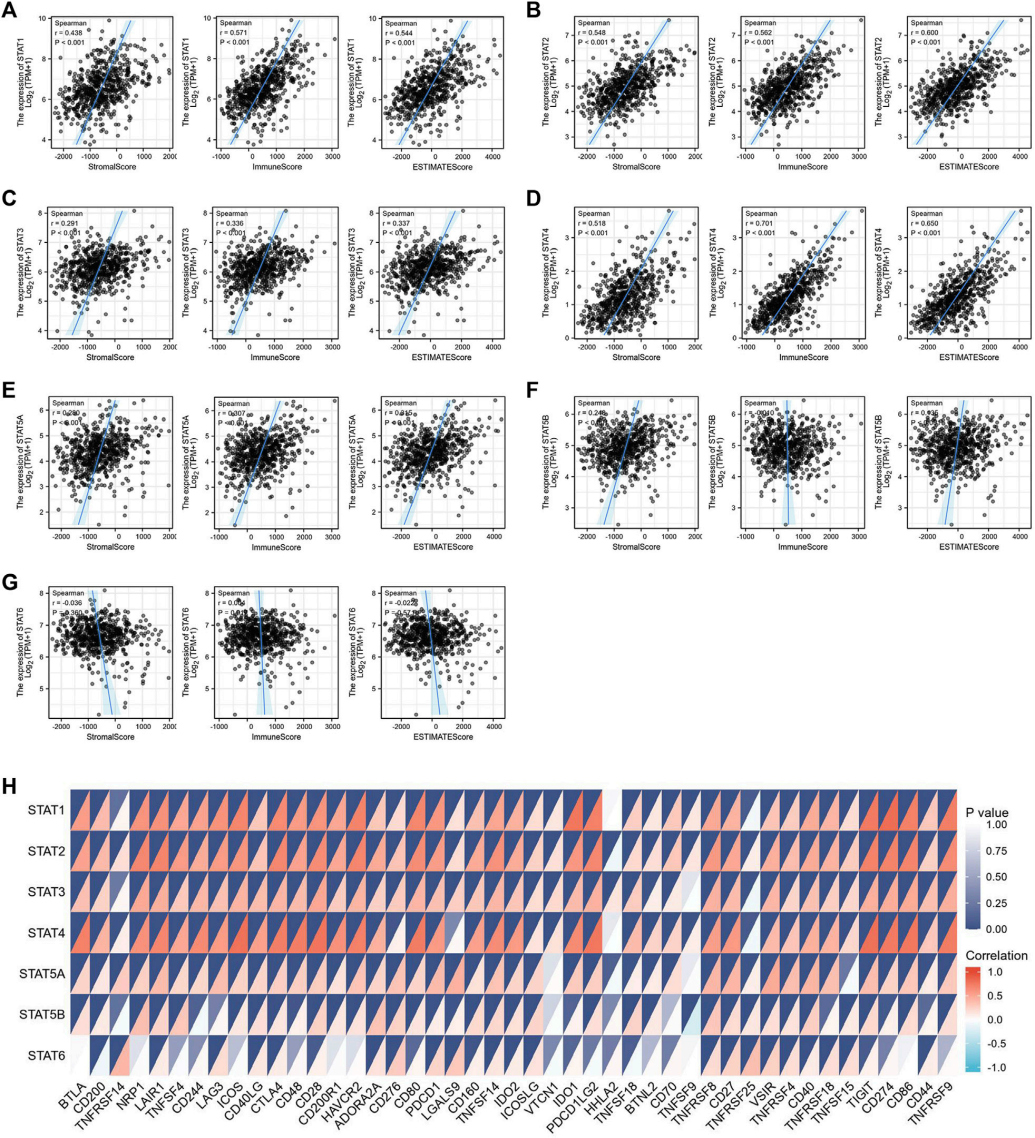
Relationship between signal transducer and activator of transcription (STAT) factor expression and stromal score, immune score, ESTIMATE score, and immune checkpoints in colorectal cancer (CRC). The correlation between the expression of **(A)** *STAT1*, **(B)** *STAT2*, **(C)** *STAT3*, **(D)** *STAT4*, **(E)** *STAT5A*, **(F)** *STAT5B*, and **(G)** *STAT6*, and stromal score, immune score, and ESTIMATE score in CRC. **(H)** Correlation heatmap of STATs with immune checkpoints in CRC.

**FIGURE 11:**
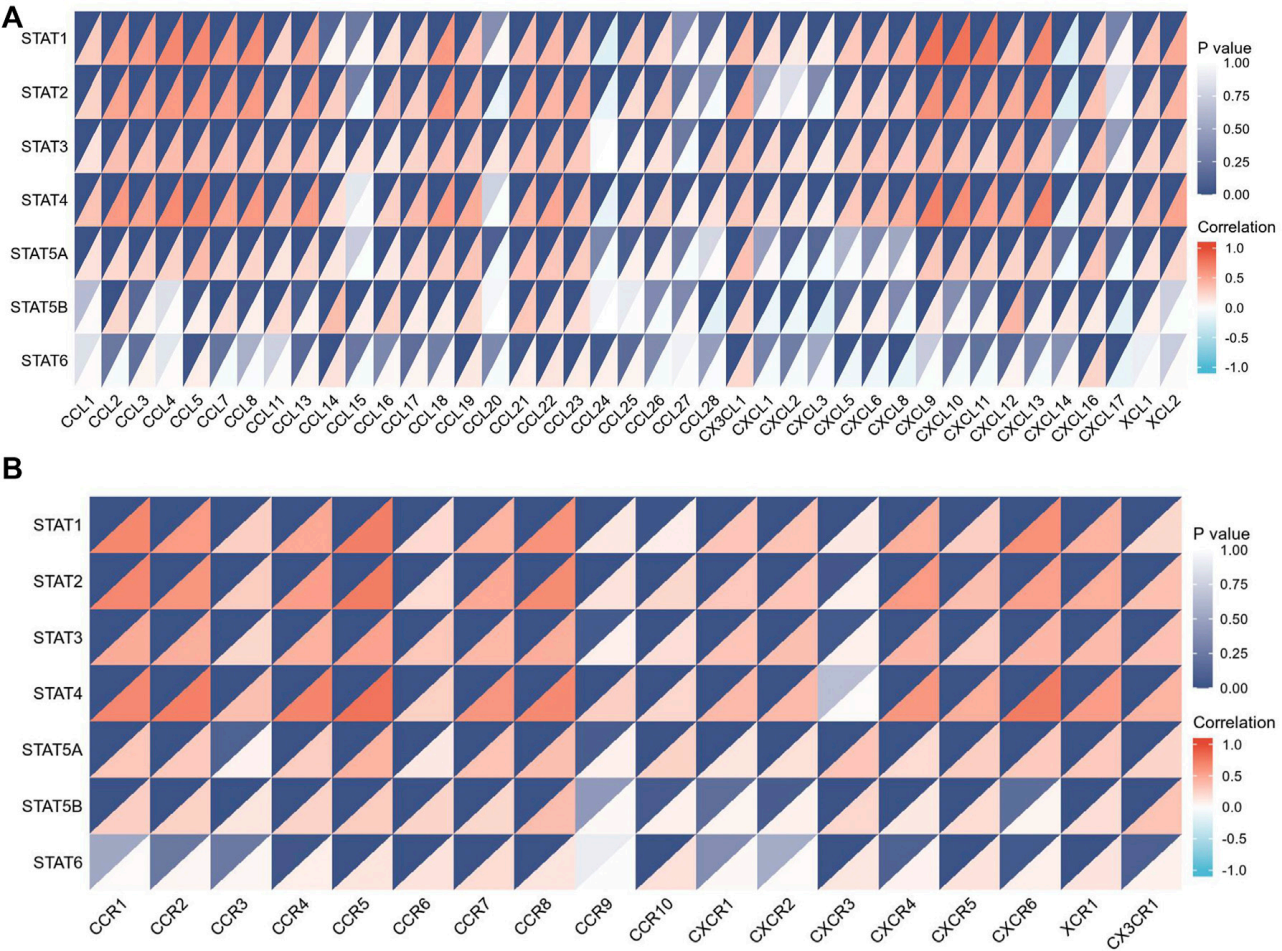
Relationship between signal transducer and activator of transcription (STAT) factor gene expression and chemokines as well as their receptors. **(A)** Correlation between STAT expression and multiple chemokines. **(B)** Correlation between STAT expression and multiple chemokine receptors.

The tumor microenvironment (TME) includes tumor cells, stromal cells, immune cells, and extracellular matrix. Stromal cells have been reported to promote tumorigenesis in many ways, and infiltration levels of different immune cells are also linked to tumor prognosis. As a result, the correlation between STAT gene expressions and stromal cells and immune cell content in CRC was subsequently evaluated. The results showed that the expressions of *STAT1*, *STAT2*, *STAT3*, *STAT4*, and *STAT5A* were positively related to stromal score, immune score, and ESTIMATE score with great significance (*p* < 0.001) (Figures 10A–E). Also, the expression of *STAT5B* was positively related to the stromal score and the ESTIMATE score, but there was no relationship between *STAT6* expression and these scores as shown in Figure 10F and Figure 10G. The correlation analysis showed that STAT gene expressions were strongly positively related to eight immune checkpoints, including CD40LG, ADORA2A, TNFSF14, ICOSLG, TNFRSF8, CD27, VSIR, and TNFRSF4 (*r* > 0, *p* < 0.001) (Figure 10H and Table 3). In addition, the expressions of STAT genes were also correlated with most chemokines and their receptors (Figure 11).

**TABLE 3:** Relationship between signal transducer and transcription (STAT) factor expression and immune checkpoints in colorectal cancer (CRC).

R	Immune checkpoint							
	CD40LG	ADORA2A	TNFSF14	ICOSLG	TNFRSF8	CD27	VSIR	TNFRSF4
*STAT1*	0.311	0.315	0.501	0.189	0.488	0.429	0.325	0.282
*STAT2*	0.326	0.483	0.618	0.257	0.576	0.497	0.460	0.372
*STAT3*	0.373	0.261	0.409	0.194	0.433	0.378	0.409	0.234
*STAT4*	0.536	0.444	0.647	0.152	0.544	0.572	0.383	0.329
*STAT5A*	0.234	0.241	0.336	0.237	0.409	0.405	0.478	0.366
*STAT5B*	0.178	0.335	0.224	0.233	0.307	0.199	0.173	0.158
*STAT6*	0.133	0.163	0.139	0.158	0.199	0.254	0.263	0.187

The *p-*value of immune checkpoints (CD40LG, ADORA2A, TNFSF14, ICOSLG, TNFRSF8, CD27, VSIR, and TNFRSF4) is less than 0.001.

**FIGURE 12:**
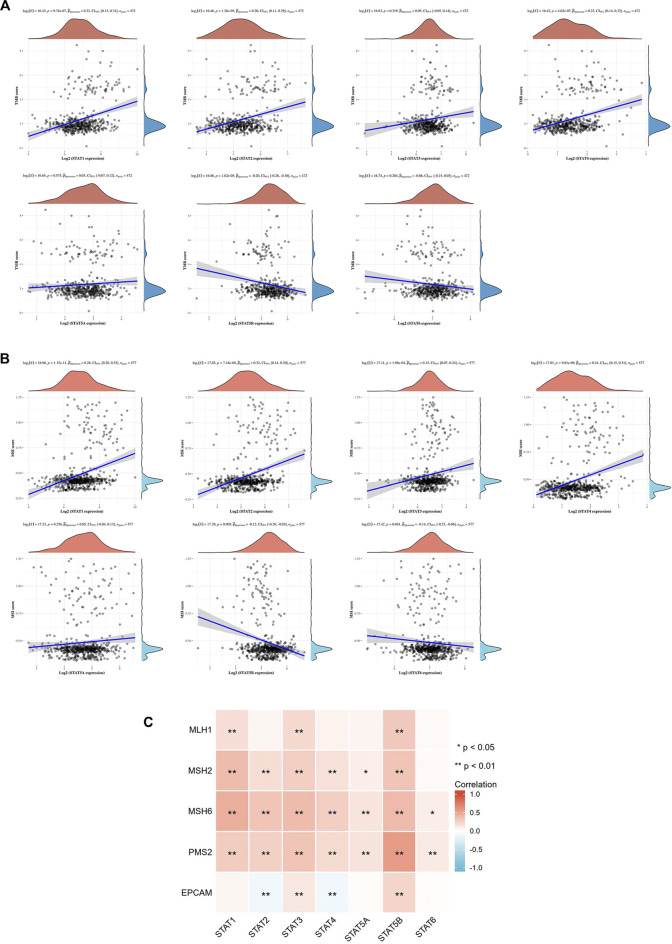
Correlation between signal transducer and activator of transcription (STAT) factor expression and TMB score, MSI score, and MMR genes. **(A)** Correlation between STAT expression and TMB score. **(B)** Correlation between STAT expression and MSI score. **(C)** Correlation heatmap of STAT expression with MMR genes in CRC.

TMB is a predictive biomarker of response for cancer patients receiving immune checkpoint blockade. The study results revealed a positive relationship between TMB score and *STAT1*, *STAT2*, and *STAT4* expression (*p* < 0.001), and a negative relationship between TMB score and *STAT5B* expression (*p* < 0.001) (Figure 12A). The MSI status is also closely linked to the response to immune checkpoint blockade, especially in patients with CRC. As a result, the correlation between STAT expression and MSI score was also assessed, and the results indicated that *STAT1*, *STAT2*, *STAT3*, and *STAT4* were positively correlated with MSI score (*p* < 0.001), whereas *STAT5B* and *STAT6* were negatively correlated with MSI score (*p* < 0.01) (Figure 12B). The relationship between STAT expression and MMR genes such as MLH1, MSH2, MSH6, PMS2, and EPCAM was evaluated using TCGA expression profile data. The results showed that the expressions of *STAT1*, *STAT2*, *STAT4*, and *STAT5A* were positively correlated with MSH2, MSH6, and PMS2 (*p* < 0.05); *STAT3* and *STAT5B* were also positively correlated with all these genes, whereas *STAT2* and *STAT4* were negatively correlated with EPCAM (*p* < 0.01) (Figure 12C).

## Discussion

Extensive studies have indicated that the abnormal regulation of STATs, especially for *STAT1/3/5*, is closely associated with the progression of various tumors, including solid tumors and hematologic malignancies, such as prostate cancer, breast cancer, CRC, and leukemias (Ferrajoli et al., 2006; Lassmann et al., 2007; Benekli et al., 2009; Gu et al., 2010; Koptyra et al., 2011). JAK/STAT signaling has also been identified as one of the key pathways affected by the majority of cancer gene mutations (Vogelstein and Kinzler, 2004). Although some studies have reported the role of certain STAT factors in cancer progression, there have been no studies that have comprehensively analyzed the role of different STATs in CRC. For the first time, bioinformatics analysis was used to investigate the transcription and translation levels, genetic variation, biological function, and molecular mechanism of STATs in CRC, as well as their correlation with prognosis, immune infiltration, and immunotherapy response.

Some studies demonstrate that activated *STAT1*, as a tumor suppressor, is lost in several types of malignant cells (Adámková et al., 2007), including breast cancer (Koromilas and Sexl, 2013), lung cancer (Chen et al., 2015), and esophageal squamous cell carcinoma (Zhang et al., 2014), and many reports indicate that high *STAT1* expression means better clinical prognosis (Widschwendter et al., 2002; Deng et al., 2012; Hosui et al., 2012). However, contradictory results have also been reported, showing that high *STAT1* expression levels are found in some cancers and correlate with poor prognosis compared to those with low expression levels, such as breast cancer, glioblastoma, lymphoma, and renal cell carcinoma (Khodarev et al., 2004; Duarte et al., 2012; Greenwood et al., 2012; Zhu et al., 2012; Arzt et al., 2014). In this study, database analysis showed that the transcription and translation levels of *STAT1* in CRC were higher than those in normal tissues, which was further verified by IHC staining. Furthermore, the expression of *STAT1* in patients with CRC was found to be significantly related to the tumor stage. A survival analysis revealed that a high *STAT1* transcription level had no correlation with the prognosis of patients with CRC but led to better OS in READ.

Previous reports suggest that *STAT2*, like *STAT1*, may play a dual role in cancer progression. Clifford et al. reported that sustained *STAT2* expression was required for interferon alpha-induced tumor-suppressive effects in skin squamous cell carcinoma cells, and the tumor-suppressive activity was also demonstrated in mice models (Clifford et al., 2003; Wang et al., 2003). The role of *STAT2* in the tumorigenesis of CRC and skin cancer has also been described (Gamero et al., 2010). The results of data analysis and IHC in this study indicated that *STAT2* was less expressed in CRC than in normal tissues at the transcription and translation level. However, *STAT2* expression in patients with CRC had no relationship with the tumor stage. A survival analysis showed that high *STAT2* expression was related to better OS and DFS in patients with CRC.

The bulk of evidence indicates that *STAT3* significantly correlates with cancer development and immune escape (Bromberg, 2002; Yu et al., 2009). Abnormal elevated *STAT3* activity has been found in a variety of hematological and solid malignancies such as acute myeloid leukemia (AML), multiple myeloma, and cancers of the bladder, head and neck, kidney, pancreas, uterus, ovary, esophagus, and breast (Chen et al., 2008; Sahu and Srivastava, 2009; Bar-Natan et al., 2012; Li et al., 2013; Suh et al., 2015; Geiger et al., 2016; Subramaniam et al., 2016; Zhang et al., 2016). In addition, high levels of phosphorylated *STAT3* expression often result in a poor prognosis in many types of cancer (Kusaba et al., 2006; Macha et al., 2011; Chen et al., 2013). However, controversial evidence has emerged showing that *STAT3* plays a negative role in the tumorigenesis of KRAS-induced lung cancer in mice models (Grabner et al., 2015). A high *STAT3* expression correlates with a better clinical outcome in patients with CRC and nasopharyngeal carcinoma (Hsiao et al., 2003; Gordziel et al., 2013). The analysis of the GEPIA dataset and IHC in this study showed that the expression level of *STAT3* in CRC was not different from that in adjacent normal tissues, but was associated with the tumor stage of patients with CRC. Survival analysis indicated that high *STAT3* expression was associated with better DFS, but did not affect OS in patients with CRC.

The exact role of *STAT4* in tumorigenesis remains unclear because high levels of *STAT4* expression have been shown to promote invasion and metastasis in gastric cancer and ovarian cancer (Zhou et al., 2014; Zhao et al., 2017), and potentially predict a favorable outcome in these cancers (Li et al., 2017; Nishi et al., 2017). The results from this study demonstrated that although there was no significant difference in *STAT4* expression between CRC tissues and normal tissues, its expression was related to tumor stage, and increased *STAT4* expression level favored OS and DFS in patients with CRC.

*STAT5*, which consists of *STAT5A* and *STAT5B*, has been reported to be implicated in multiple malignancies. Hassel et al. (2008) reported that STAT5, a tumor promoter, aided cell proliferation and survival in melanoma by activating the antiapoptotic protein Bcl-XL. It also has been suggested that activated *STAT5* signaling promotes tumor growth, invasion, and epithelial-to-mesenchymal transition (EMT) in squamous cell carcinoma of the head and neck (SCCHN), leading to resistance to chemotherapy (Koppikar et al., 2008). Furthermore, highly activated *STAT5* results in poor prognosis in patients with prostate cancer (Li et al., 2005). However, increasing evidence suggests that *STAT5* proteins also regulate the activities of tumor suppressor genes, and activated *STAT5* is associated with a favorable prognosis in patients with breast cancer and nasopharyngeal cancer (Hsiao et al., 2003; Nevalainen et al., 2004). This study found that *STAT5A* and *STAT5B* were significantly less expressed in CRC compared to normal tissues at both the mRNA and protein levels. In patients with CRC, *STAT5B* had a significant relationship with tumor stage and exerted a favorable effect on OS and DFS but *STAT5A* did not.

Some studies have reported that activated *STAT6* signaling is important for IL-4 and IL-13-induced EMT and CRC cell aggressiveness (Cao et al., 2016; Chen et al., 2018). Furthermore, inhibition studies have indicated that targeting *STAT6* signaling can suppress tumor growth and metastasis in gastric cancer (Lu et al., 2018). The data analysis revealed that there was no significant difference in *STAT6* expression between CRC tissues and normal tissues, and *STAT6* expression level did not affect tumor stage and prognosis.

The analysis outcomes showed that there was a high mutation rate (43%) of STATs in patients with CRC, and the genes with the highest and lowest mutation rates in STATs are *STAT5B* (15%) and *STAT4* (7%), respectively. Besides, the mutation of STATs had some relationship with certain types of immune cells infiltration, but showed little effect on prognosis in patients with CRC. The co-expression relationships between different STAT members in patients with CRC indicated that these factors may play a synergistic role in the progression of CRC. To evaluate the potential interactions between STAT factors and their neighboring genes at different levels, GGI and PPI networks were constructed. GO and KEGG enrichment analyses were also performed to explore the functions of STATs and their similar genes, which are primarily related to lymphocyte activation, cytokine-mediated signaling pathway, positive regulation of immune response, and T-cell receptor signaling pathway. These pathways have a close relationship with the immune system, indicating the possible role of STATs in regulating the tumor immune microenvironment.

The study results indicated that STAT transcription levels were closely correlated with levels of immune infiltration in CRC. *STAT1/2/3/4* expressions were positively correlated with the infiltrations of B cells, CD8^+^ T cells, CD4^+^ T cells, macrophages, neutrophils, and dendritic cells. The expressions of *STAT5A/5B/6* were positively associated with infiltrations of CD4^+^ T cells, macrophages, neutrophils, and dendritic cells, and *STAT5A* expression was also positively correlated with the infiltration of B cells. Also, the expressions of *STAT1/2/3/4/5A* were positively correlated with the stromal score, immune score, and ESTIMATE score with great significance. The close relationship between the expression levels of STATs and infiltration of multiple types of immune cells further demonstrates that STATs may be the regulators of tumor immunity in CRC.

Another significant finding of this study is that STATs may be possible predictors of treatment response to immunotherapy in CRC because of the close relationship between STATs and indicators associated with immunotherapy response, such as immune checkpoint genes, TMB score, MSI score, and MMR genes. Our results showed that seven STAT members were positively co-expressed with eight immune checkpoints including CD40LG, ADORA2A, TNFSF14, ICOSLG, TNFRSF8, CD27, VSIR, and TNFRSF4. From literature searches, it was found that CD40LG and CD27, both of which are stimulatory immune checkpoints, are more likely to be used clinically. Extensive studies have been conducted to investigate the underlying mechanism by which CD40LG and CD27 modulate tumor immunity. Additionally, various types of strategies targeting them, such as agonistic/antagonistic monoclonal antibodies, cellular vaccines, and protein antagonists, have been developed and demonstrated to be safe and efficacious in early clinical trials (Starzer and Berghoff, 2020; Tang et al., 2021). Coincidentally, the factor that is most closely related to both CD40LG and CD27 is *STAT4* based on their correlation coefficient (Table 3). It was discovered that almost all the STAT factors were positively co-expressed with certain MMR genes such as MSH2, MSH6, and PMS2. A positive relationship between the TMB score and STAT1/2/4 expression and a negative relationship between the TMB score and *STAT5B* expression were observed. Moreover, *STAT1/2/3/4* expression had positive correlations with the MSI score, but *STAT5B* and *STAT6* had a negative relationship with the MSI score.

This study indicates that the expression levels of *STAT2/5A/5B* are downregulated in CRC and could inhibit the initiation and development of CRC. The close relationship between the CRC stages and expression levels of *STAT1/3/4/5B* reveals their potential as molecular biomarkers for tumor stage classification. Moreover, the abnormal expressions of *STAT2/4/5B* have the potential to be used as prognostic predictors in patients with CRC. Besides, the strong association between the expression of STAT and infiltration of multiple types of immune cells in CRC, including B cells, CD8^+^ T cells, CD4^+^ T cells, macrophages, neutrophils, and dendritic cells, demonstrates that STATs may play a role in the regulation of CRC tumor immunity. More importantly, the significant correlation between STAT expressions and immunotherapy response-associated indicators showed that they had the potential to predict response to immunotherapy in patients with CRC and could be used to assist the physician in deciding on a therapeutic regimen. These findings aid in better understanding the molecular landscape of CRC progression, providing new prognostic biomarkers, and promoting the development of more immunotherapeutic strategies for patients with CRC. However, further investigations are still needed to validate the results of this study to facilitate the clinical application of STATs as therapy targets, prognostic biomarkers, and immunotherapy predictors in CRC.

## Data Availability

The original contributions presented in the study are included in the article/Supplementary Material; further inquiries can be directed to the corresponding authors.
