# Supplementary figures and images for: Comprehensive analysis of the prognostic and immunotherapeutic implications of STAT family members in human colorectal cancer
Source: Front Genet. 2022 Aug 19;13:951252. doi: 10.3389/fgene.2022.951252 (PMC9437353; doi:10.3389/fgene.2022.951252)

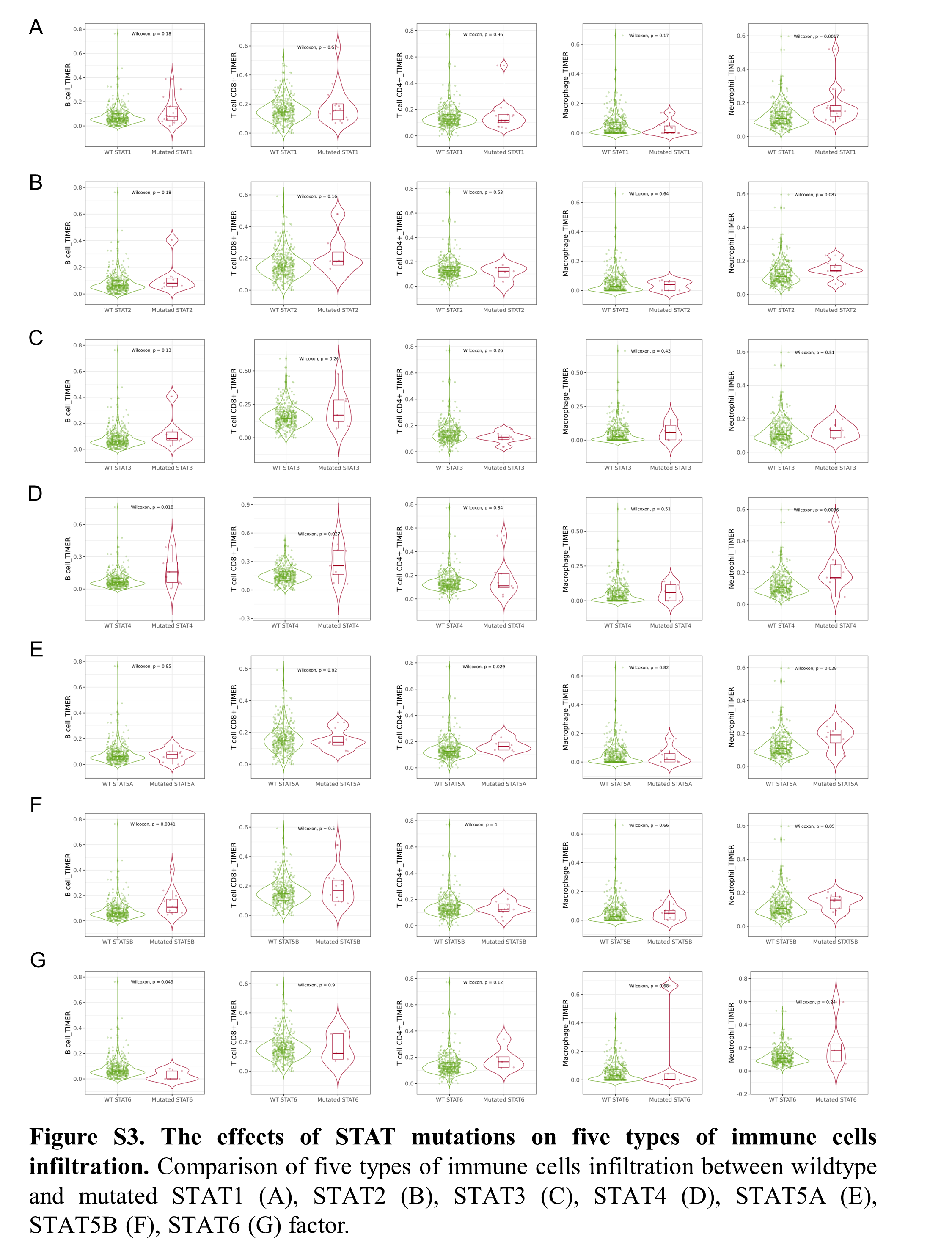

Supplement: Supplementary file 1 [file Image3.TIF]

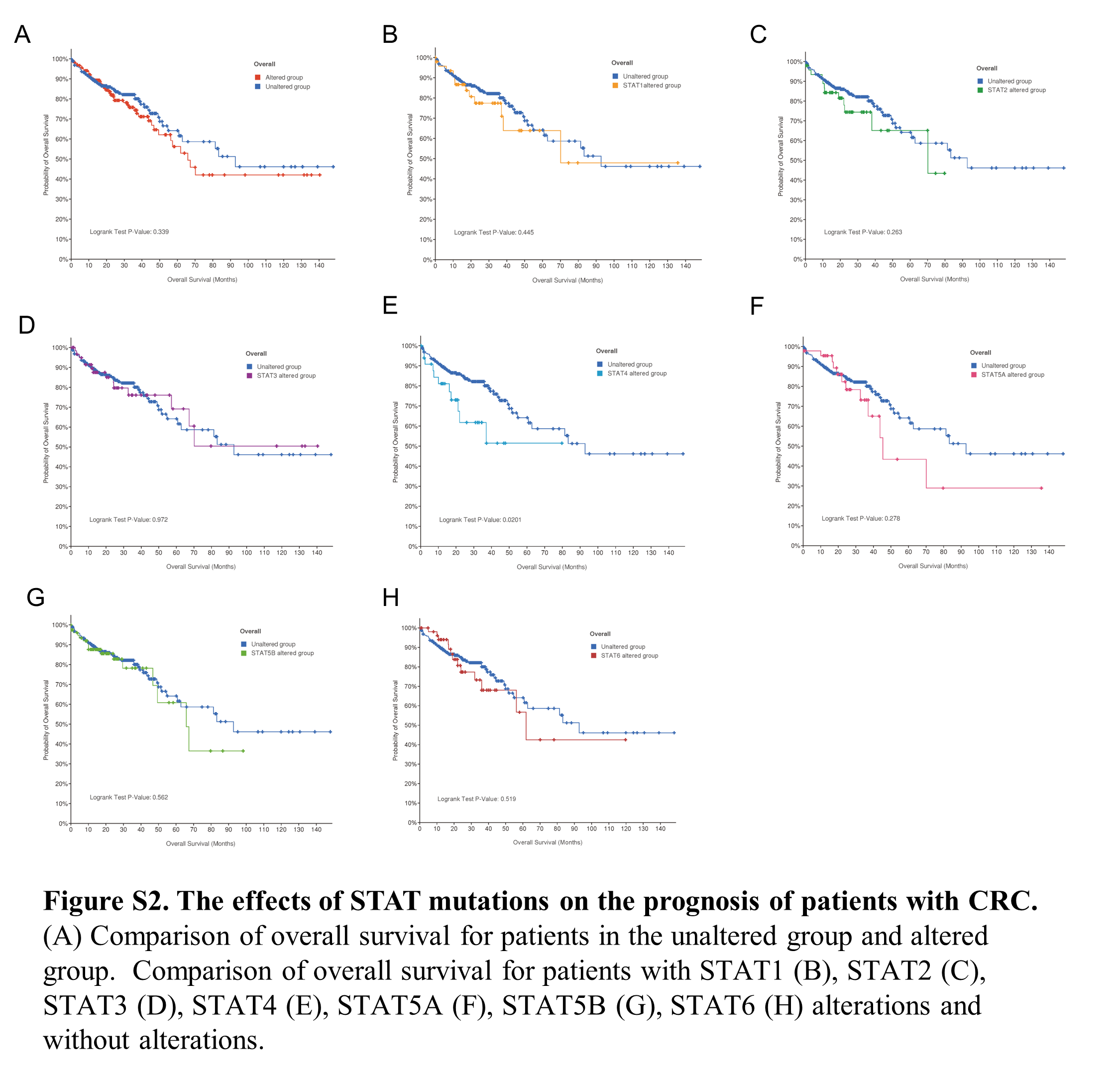

Supplement: Supplementary file 2 [file Image2.TIF]

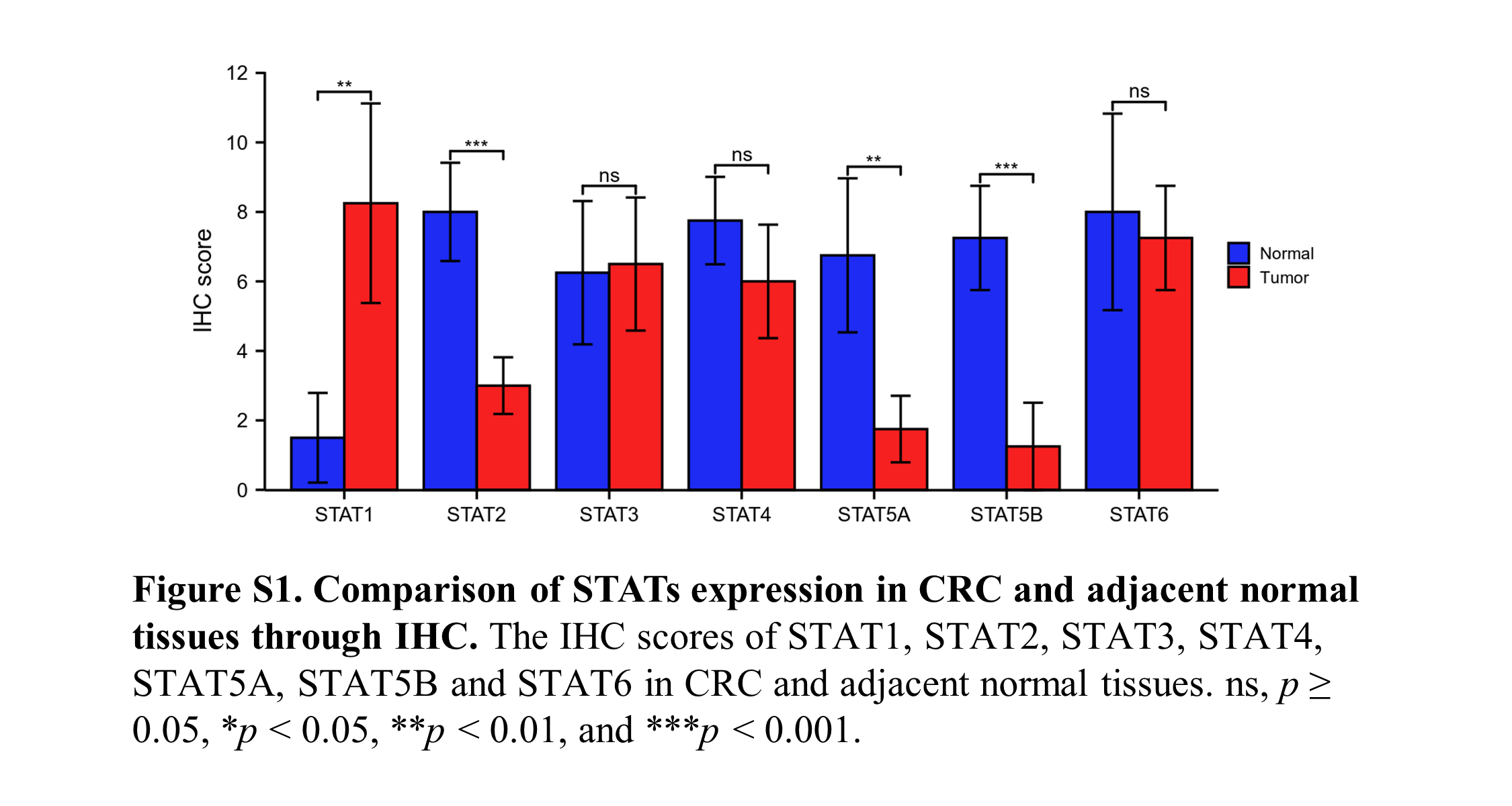

Supplement: Supplementary file 3 [file Image1.TIF]
